# The path to function: using eye-tracking in a real-world task to understand the performance gap for people with severe mental illness

**DOI:** 10.1192/j.eurpsy.2022.1894

**Published:** 2022-09-01

**Authors:** S. Regev, N. Josman, A. Mendelsohn

**Affiliations:** 1 University of Haifa, Occupational Therapy, Haifa, Israel; 2 University of Haifa, Neurobiology, Haifa, Israel

**Keywords:** Translational research, Grocery shopping, SMI, Executive function

## Abstract

**Introduction:**

Individuals with severe mental illnesses (SMI) often present the knowledge about a task but in real-time do not perform it fully, or not as efficient as planned. This performance gap may be explained by difficulties with Executive Functions (EF).

**Objectives:**

The aim of the presentation is to describe how people with and without SMI experience and perform grocery task. This, with considering this path from several directions including the subjects’ point of view using eye-tracking device during task performance.

**Methods:**

Forty-three individuals had answered questions in regards to their shopping habits and performed the Test of Grocery Shopping Skills (TOGSS). The actual performance was accompanied by wearing an eye-tracking device which recorded the behavior and eye movement. We hypothesized that significant differences will be found between people with SMI and controls both in the routine grocery habits and in observed performance.

**Results:**

No significant differences in age or gender. The groups differed significantly only in education, with the SMI group having fewer years of education. As a weekly routine, SMI subjects perform less frequent shopping (40%) than control group subjects (67%). TOGSS sub-outcomes indicated performance efficiency (time and redundancy) were significantly higher in the research group than in the matched control group (p <.01), with the SMI group spending a longer time performing the task and entering more aisles than required – redundancy.

**Conclusions:**

These preliminary findings indicate that individuals with SMI spend more time dwelling while selecting ingredients. Besides the path in the supermarket, it might explain their performance in other everyday activities.

**Disclosure:**

No significant relationships.

